# In Situ Grown Mesoporous Structure of Fe-Dopant@NiCoO_X_@NF Nanoneedles as an Efficient Supercapacitor Electrode Material

**DOI:** 10.3390/nano13020292

**Published:** 2023-01-10

**Authors:** Yedluri Anil Kumar, Ganesh Koyyada, Dasha Kumar Kulurumotlakatla, Jae Hong Kim, Md Moniruzzaman, Salem Alzahmi, Ihab M. Obaidat

**Affiliations:** 1 Department of Chemical & Petroleum Engineering, United Arab Emirates University, Al Ain 15551, United Arab Emirates; 2 National Water and Energy Center, United Arab Emirates University, Al Ain 15551, United Arab Emirates; 3 Department of Chemical Engineering, Yeungnam University, 214-1, Daehak-ro 280, Gyeongsan 712-749, Gyeongbuk-do, Republic of Korea; 4 Graduate School of Convergence Science, Pusan National University, San 30 Jangjeon-dong, Geumjeong-gu, Busan 609-735, Republic of Korea; 5 Department of Chemical and Biological Engineering, Gachon University, 1342 Seongnam-daero, Seongnam-si 13120, Gyeonggi-do, Republic of Korea; 6 Department of Physics, United Arab Emirates University, Al Ain 15551, United Arab Emirates

**Keywords:** capacitive contribution, Fe-dopant, nanoneedles structure, NiCoO_x_, specific capacitance

## Abstract

In this study, we designed mixed metal oxides with doping compound nano-constructions as efficient electrode materials for supercapacitors (SCs). We successfully prepared the Fe-dopant with NiCoO_x_ grown on nickel foam (Fe-dopant@NiCoO_x_@NF) through a simple hydrothermal route with annealing procedures. This method provides an easy route for the preparation of high activity SCs for energy storage. Obtained results revealed that the Fe dopant has successfully assisted NiCoO_x_ lattices. The electrochemical properties were investigated in a three-electrode configuration. As a composite electrode for SC characteristics, the Fe-dopant@NiCoO_x_@NF exhibits notable electrochemical performances with very high specific capacitances of 1965 F g^−1^ at the current density of 0.5 A g^−1^, and even higher at 1296 F g^−1^ and 30 A g^−1^, respectively, which indicate eminent and greater potential for SCs. Moreover, the Fe-dopant@NiCoO_x_@NF nanoneedle composite obtains outstanding cycling performances of 95.9% retention over 4500 long cycles. The improved SC activities of Fe-dopant@NiCoO_x_@NF nanoneedles might be ascribed to the synergistic reactions of the ternary mixed metals, Fe-dopant, and the ordered nanosheets grown on NF. Thus, the Fe-dopant@NiCoO_x_@NF nanoneedle composite with unique properties could lead to promising SC performance.

## 1. Introduction

These days, there is a growing insistence on greener and more renewable energy sources, including hydro-energy and solar power, to replace fossil fuel applications, which have negative environmental effects [1,2]. Superior electrochemical storing devices that are safe and eco-friendly with energy durability are therefore needed [3,4,5]. Supercapacitors (SCs), as currently recommended energy-storing equipment, have been vitally improved for electric motors, portable electrical devices, and networks through virtue of great power densities, ultrafast charging/discharging, and prolonged life cycles [6,7]. It is well known that operational sample electrodes are important components of SCs. Thus, three representative anode electrode materials—denoted graphite electrodes [8,9,10], transitional metal compounds [11,12,13], and conducting polymers [10,11]—have been very often reported. In general, SCs lag behind fuel cells and batteries in terms of electrochemical energy density, which limits their practical utility for near-future applications [14,15]. Until now, various electrode bases that include oxides [16,17,18], carbons [19,20], and conducting polymers [21,22,23] have been exerted.

Developing the specific discharge capacitances (C) is regarded as a feasible path to effectively improving the energy densities of SC devices [24,25]. Because of the inconsistency of electrochemical storing principles, pseudocapacitors (PCs) provide superior energy densities compared to electric double-layer capacitors (EDLCs). Binary metal electrodes have obtained much consideration because of their eco-friendliness, easier fabrication, and great energy storage performances compared with single metal oxides. NiCo_2_O_4_ is composed of spinel nano-structures through face-centered cubic architecture with variant stacks of oxygen octahedra (NiO_6_, CoO_6_) and tetrahedra (CoO_4_) [26], which were reported as the foremost SCs. These materials have great conductivity, ultra high specific capacitances, and outstanding capacity retention comparable to the single-metal counterpart components NiO and Co_3_O_4_ [27,28]. Nevertheless, the single NiCo_2_O_4_ electrode is still limited by its low rate capability and slow cycles, manifesting its insufficient electro-kinetic characteristics and low specific region resulting from its intrinsic demerits [29]. Very recently, some scientists reported and designed NiCo_2_O_4_ sheet sample materials for SC properties. For instance, Wang et al. designed NiCo_2_O_4_@NiCo_2_O_4_ nano-cone types with notable specific capacitance of 2045 F g^−1^ and high energy density of 82.7 Wh·kg^−1^ at 351 W·kg^−1^ [30]. Moreover, Gao et al. presented NF@NiCo_2_O_4_ nano-feather composites with C of 1797 F g^−1^ and energy density of 53.8 Wh·kg^−1^ at 802 W·kg^−1^ [31]. In addition, NiCo_2_O_4_ mesoporous samples have been prepared by J. Acharya et al. with C of 790 F g^−1^ and energy density of 42.4 Wh·kg^−1^ with power density of 746.2 W·kg^−1^ [32]. To overcome the conductiviy drawbacks, metal transition elements’ dopant procedures have been considered as efficient, and serious approaches have been made by supplying defects, tuning the intrinsic electrical conductivity, and supplying many holes to condense the pathways for ionic diffusion while the redox reaction occurs [28]. Among the transition elements, cobalt (Co) has been seen as an essential source for SC application owing to its higher capacity results, cheaper prices, innate abundance, and eco-friendliness. The iron ion (Fe^3+^) has been selected as the dopant element to boost electrical kinetics with conductivity characteristics, raise the electrochemically activated sites, and accelerate the redox process for obtaining higher electrochemical performances [28,33,34]. Due to this underlying reason, the usage of the Fe-based metals on the current collector is considered an effective strategy to enhance capacity performance, leading to high energy density, increased capacity distribution, and stable structures. In addition, nickel foam-based nano-composites are widely employed to improve electrochemical stability for long discharge cycles [35], using their major specific area, excellent conductivity characteristics, and outstanding thermal stability [36]. Still, several synthesis methods are unexplored in this field. However, by identifying suitable synthesis methods for a specific application (such as energy storage), one can achieve improved supercapacitor performance. To this end, we have adopted this concept and validated the role of the hydrothermal method and temperature in depositing ternary metal oxides such as cobalt, nickel, and copper as cations.

Herein, we synthesized a unique ternary mixed metal of Fe-dopant@NiCoO_x_@NF nanoneedles with enhanced electrochemical activities for SCs through a hydrothermal route followed by an annealing process. To understand the synergistic catalytic effects, we have presented seven different ratios of Fe to study SC performance. The most synergistic effects of the two metals enable Fe-dopant@NiCoO_x_@NF nanoneedle composites to provide high specific discharge times, notable rate capabilities, conserable conductivity, and stability in cycles while employed in SCs. Fe-dopant@NiCoO_x_@NF nanoneedles possess hierarchical sheet constructions that could supply plentiful paths for better ion transportation. Impressively, the sheet-like Fe-dopant@NiCoO_x_@NF composite delivered excellent super-capacitive behavior, including capacitance of 1965 F g^−1^ with 95.97% superb rate capabilities even after 4500 long cycles that were derived from the synergetic contributions of Ni, Fe, and Co oxides.

## 2. Experiment

### 2.1. Materials

All the reagents are analytically graded and used with no further purification. Typical nickel foam substrates were successfully pretreated with aqueous 3 M HCl solution, ethanol, and DI water before utilization.

### 2.2. Fabrication of the Fe-dopant@NiCoO_x_@NF Nanoneedles

Initially, 1 mmol Co(NO_3_)_2_·6H_2_O and 0.5 mmol Ni(NO_3_)_2_·6H_2_O were added to 150 mL deionized water (DI) with constant stirring for 45 min to give a light pink precursor. In addition, Fe-dopant reagent and 43 mg FeCl_3_·6H_2_O were were added to the mixture. Then, 70 mg PVP and 350 mg urea were also added to the above precursors. Light pink colored solutions were formed through vigorous stirring. The described solution was put under ultrasonication for 25 min, moved to an autoclave, and optimized at 110 °C for 15 h. Afterwards, the procured black powders were cleansed with DI water and ethanol various times and dried at 50 °C overnight.

### 2.3. Fabrication of the NiCoO_x_@NF Nanowires

The NiCoO_x_@NF nanowires were prepared using an easy hydrothermal technology with the combination of an annealing process. Additionally, pure NiCoO_x_ nanowires without Fe-dopant sources were also produced to be comparable with the Fe-dopant@NiCoO_x_@NF samples. Eventually, the accumulated precursors were optimized at 350 °C for 180 min with ramping speeds of 3 °C·min^−1^ to obtain NiCoO_x_@NF nanowires and Fe-dopant@NiCoO_x_@NF nanoneedles with abundant nanosheets. The mass loading of active material NiCoO_x_@NF nanowires and Fe-dopant@NiCoO_x_@NF on Ni foam substrate was calculated to be 4.6 mg cm^−2^ and 6.9 mg cm^−2^ by subtracting the mass of bare Ni foam substrate from the mass of active material loaded onto Ni foam.

### 2.4. Measurements and Characterizations

The as-synthesized product sample was analyzed by X-ray diffraction (XRD, Bruker D8 Advance, Bruker AXS LTD., Busan, Republic of Korea) with Cu K radiation (1.5406 Å), and the sample was scanned in the 2q range from 10° to 90° in steps of 0.02° with a voltage of 40 kV and a current of 200 mA using a high resolution transmission electron microscope (TEM, JEM-2100F, JEOL LTD., Busan, Republic of Korea) and a field emission scanning electronic microscope (FE-SEM, JSM-7800F, JEOL LTD., Busan, Republic of Korea) instrumented with energy-dispersive spectra (EDS) and X-ray photoelectronic spectroscope analyzers with an energy analyzer (XPS; ESCCALAB 250Xi, Thermo Scientific LTD., Busan, Republic of Korea). Brunauer–Emmett–Teller (BET) was recorded at 77 K to examine the specific surface area and pore size utilizing a Micromeritics ASAP 2010 (Busan, Republic of Korea) absorption analyzer.

### 2.5. Electrochemical Measurements

A qualitative 3-electrode setup was created in 3 M KOH aqueous electrolytes, and all the analyses were maintained on electrochemical workstations (Bio-Logic, SP-150, Busan, Republic of Korea). The as-fabricated electrodes, Ag/AgCl, and platinum (Pt) foil electrodes were proposed as the working electrode, reference electrode, and counter electrodes, respectively. The CV measurements were conducted at different scan rates ranging from 5 to 100 mV s^−1^ within the potential window of 0–0.4 eV. The GCD measurement was carried out at different current densities ranging from 1 to 20 A g^−1^. The EIS measurements were carried out using Nyquist plots in the frequency range of 0.001 Hze100 KHz. Therefore, GCD results from varying current densities were procured to quantify the specific capacitances of electrodes. The specific capacitances were measured according to the following equation [37,38]:

*C* = (*I* × Δ*t*)/(*m ×* Δ*V*) (1)

wherein *I* is the current density, *t* is the discharge time, Δ*V* is the window range for the GCD procedures, and *m* denotes the mass of the samples.

## 3. Results and Discussion

**Figure 1:**
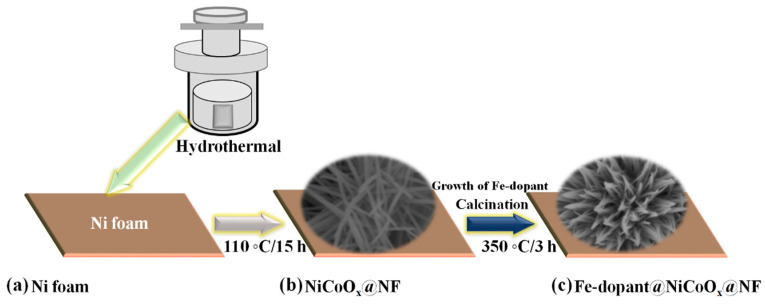
Schematic illustration of the synthesis of Fe-dopant@NiCoO_x_@NF nanoneedle composite.

The synthesis process of the unique Fe-dopant@NiCoO_x_@NF nanoneedle composite is demonstrated in Figure 1. Initially, the vertical NiCoO_x_ nanoparticles composed of nickel foam were successively organized and produced by the hydrothermal route. After that, the Fe-dopant was well decorated and dispersed on NiCoO_x_ nanowires and on nickel foam and was successively prepared through facile hydrothermal followed by annealing.

**Figure 2:**
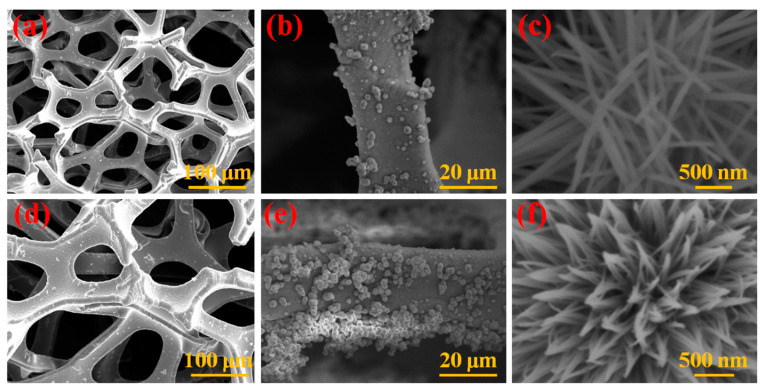
Low- and high-magnification FE-SEM images of the (**a**–**c**) NiCoO_x_@NF nanowires; (**d**–**f**) Fe-dopant@NiCoO_x_@NF nanoneedle composite.

The micro-morphologies and the structural characteristics of the as-designed NiCoO_x_@NF and Fe-dopant@NiCoO_x_@NF product samples were analyzed using an FE-SEM microscope. As shown in Figure 2a, the NiCoO_x_ nanowires were vertically developed and homogeneously formed on the nickel foam skeleton structure. Figure 2b,c demonstrated that the NiCoO_x_ morphologies possess a nanowire formation with steady sizes between 3–5 μm formulated from abundant nanowires grown on the NF skeleton surface area. As demonstrated in Figure 2c, all nanowires consist of diameters of ranges between 100 and 200 nm with a span length of 1–2 μm. Impressively, all wire-shaped structures are attributed to the many minor internally connected crystals that possess uniform sizes (Figure 2c). Figure 2d–f corresponds to the Fe-dopant@NiCoO_x_@NF nanoneedle composite in FE-SEM images. The influence of Fe-dopant on NiCoO_x_ morphology is clearly seen, and the diameters of the nanowires gradually decreased to be in the range of 40–60 nm, while the lengths were preserved due to the effect of the Fe-dopant on the NF foam substrate (Figure 2f). It is seen that the mesoporous property of NiCoO_x_ was forcibly affected by the Fe-dopant. The Fe-dopant@NiCoO_x_@NF nanoneedle composite possesses rough interfaces that substituted for the smoother surfaces of the nanoneedles after annealing.

**Figure 3:**
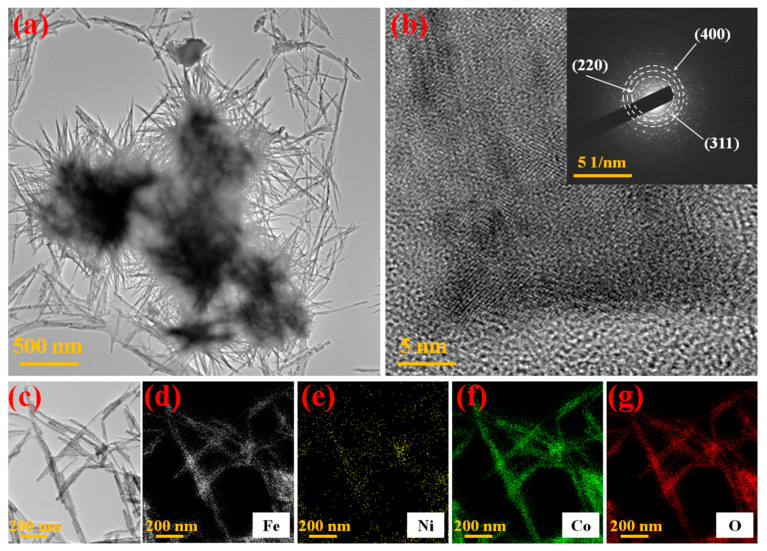
Fe-dopant@NiCoO_x_@NF nanoneedle composite sample: (**a**) TEM images, (**b**) HRTEM images (inset; SAED of the sample), and (**c**–**g**) TEM-EDS mapping of Fe, Ni, Co, and O.

TEM was used to study the microstructure of the as-developed unique composites. It can be clearly identified in Figure 3a,b that the formation of individual particles is explicit and is similar to that found in FE-SEM, with regular particles 3–5 μm in size whose diameters were around 30 to 50 nm. Impressively, these nanoparticles can be attributed to many uniform crystals, resulting in their mesoporous behavior. Therefore, the TEM results are in agreement with the SEM outcomes. The hierarchical and novel NiCoO_x_@NF nanowire microstructures and their successive ornamentation using Fe-dopant nanosheets could boost the electrical kinetics and the active sites. The selected area electron diffraction (SAED) image (shown in the inset of Figure 3b) displays multiple diffraction rings with varied diameters that are indexed with the (220), (311), and (400) phases of the NiCo_2_O_4_ spinel [25]. The SAED results reveal the polycrystalline characteristics of the sample. To further distinguish the elemental distributions found in the synthesized Fe-doped@NiCoO_x_@NF nanosheets, EDS maps were also created, as demonstrated in Figure 3c–g. EDS mappings of the sample showed that Fe, Ni, Co, and O elements uniformly covered the entire nanoneedle, indicating that Fe-dopant particles were successively decorated on NiCoO_x_.

**Figure 4:**
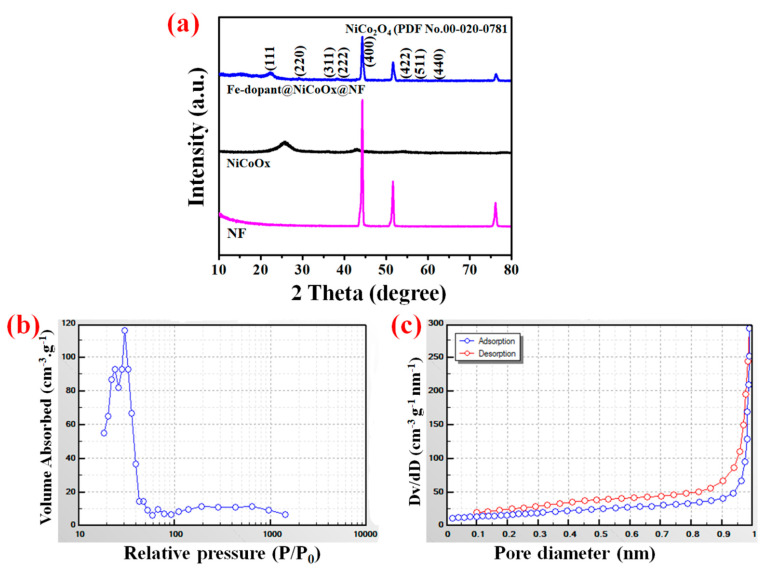
The NF, NiCoO_x_@NF, and Fe-dopant@NiCoO_x_@NF samples: (**a**) XRD pattern of the designed electrodes, (**b**) nitrogen absorption–desorption isotherm, and (**c**) the distribution of pore size analysis of the Fe-dopant@NiCoO_x_@NF composite sample.

XRD was used to investigate the phases of NF, NiCoO_x_@NF, and Fe-dopant@NiCoO_x_@NF samples. As depicted in Figure 4a, the diffraction peaks allocated at 2θ values were well ascribed to the (220), (311), (222), (400), (422), (511), and (440) spaces of the spinel NiCo_2_O_4_ angle (JCPDS No.00-020-0781) [39]. Moreover, the two representative angles at 44.53°and 51.68° could be attributed to (111) and (200) phases of NF crystals (JCPDS card no. 01-089-7128) [40,41]. The XRD patterns do not display any peaks of other phases, thus manifesting the unalloyed phases of NiCo_2_O_4_. These characteristics can affect the electrochemical performances of NiCo_2_O_4_ composites via the formation of sufficient electro-active sites for the redox mechanism procedures [29].

The distribution of pores and surface areas of the designed NiCoO_x_-based electrode samples were extensively investigated by the BET. The N_2_ absorption–desorption isotherms are shown in Figure 4b. All the fabricated electrodes displayed type-IV hysteresis with a loop at high pressure that indicates the mesoporous behaviors. The BET surface area of Fe-dopant@NiCoO_x_@NF was detected to be around 118.6 m^2^·g^−1^. Hence, the electrochemically active sites were distinctly improved because of the Fe-dopant. From the BJH image, as depicted in Figure 4c, the pore distribution sizes of the Fe-dopant@NiCoO_x_@NF composite sample displayed mesoporous distribution characteristics [42]. As illustrated in Figure 4c, the Fe-dopant@NiCoO_x_@NF sample consists of large meso/micro-pore volumes.

**Figure 5:**
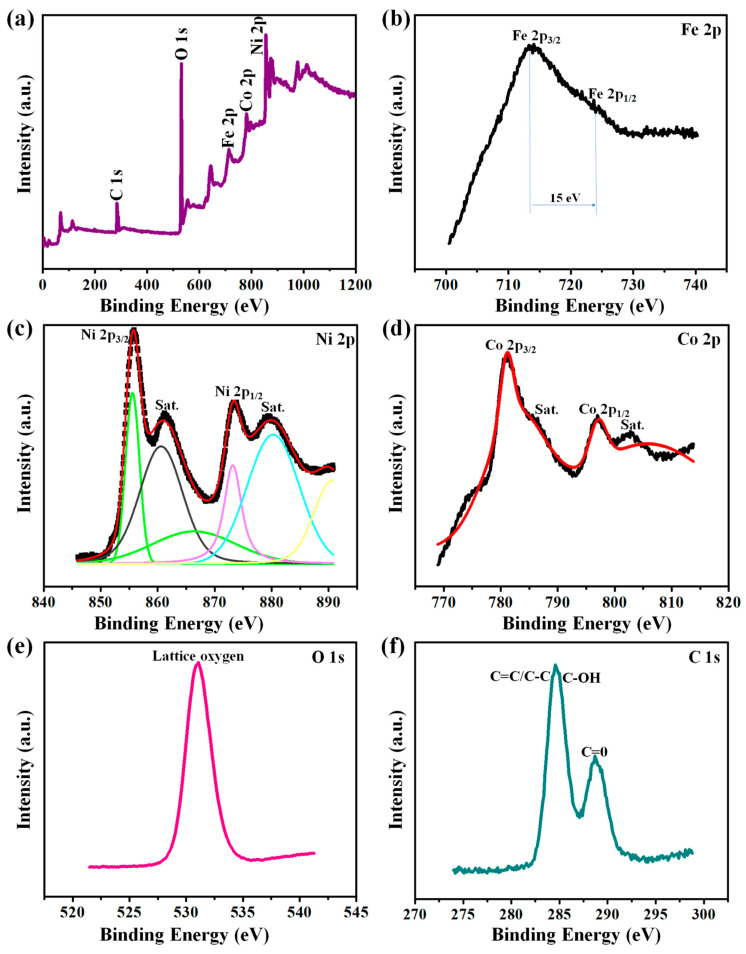
XPS spectrum of Fe-dopant@NiCoO_x_@NF nanoneedle composite: (**a**) XPS survey spectrum, (**b**–**e**) high-resolution spectrum of (**b**) Fe 2p, (**c**) Ni 2p, (**d**) Co 2p, (**e**) O 1s, and (**f**) C1s.

The comprehensive survey of XPS spectra of the Fe-dopant@NiCoO_x_@NF composite sample that were ascribed to Fe 2p, Ni 2p, Co 2p, O1s, and C1s are shown in Figure 5a. Various chemical states were extensively analyzed, and the outcomes are depicted in Figure 5b–f. The elevated spectra of Fe 2p signals can be manually deconvoluted into two existing species, Fe 2p_1/2_ (at 726 eV) and Fe 2p_1/2_ (at 710.5 eV), proving that Fe^3+^ ions were successively doped in the spinel species of the NiCo_2_O_4_ sample and outright oxidational states [28,43]. The Ni 2p spectra (Figure 5c) of elevated resolutions possess 2 sub-peaks at 855.5 and 873.4 eV, corresponding to Ni 2p_3/2_ and Ni 2p_1/2_ spin-orbit doublets, respectively. These spin-orbit deconvolution characteristics revealed the existence of 2 shake-up satellite peaks at 880.3 and 861.4 eV. All the spin-orbits and shake-up satellite signals were closed to Ni^3+^ and Ni^2+^. Moreover, the Co 2p spectra deconvolution confirmed the existence of 2 spinal-orbit doublets, Co 2p_1/2_ (at 795.4 eV) and Co 2p_3/2_ (at 780.6 eV). These spinal-orbit doublets consist of 2 supplemented shake-up species at 803.3 and 786.9 eV, corresponding to Co^2+^ and Co^3+^ (shown Figure 5d), respectively. Additionally, the sub-peaks at 794.6 and 779.1 eV are associated with Co^2+^, and those at 796.1 and 780.4 eV were related to Co^3+^. The existence of Ni^2+^, Ni^3+^, Co^2+^, and Co^3+^ peaks was also reported in recent studies [32]. In addition, the elevated fitting of O1s spectra (Figure 5e) revealed the occupancy of one strong peak. The main one at 529.6 eV was ascribed to the metal binding oxides [44] that consist of the oxygen defection (OH^−^ groups) site in the Fe-dopant@NiCoO_x_@NF interface. The C1s elevated spectra (Figure 5f) were further examined to reveal the interactions among the Fe-dopant NiCo_2_O_4_ and NF and to prove the reduction degrees of NF. In conclusion, all XPS spectra proved the existence of Fe^3+^, Ni^3+^, Ni^2+^, Co^2+^, Co^3+^, and O^2−^, which agree well with the analyzed phases of the Fe-dopant@NiCoO_x_@NF nanoneedle composite [45].

### Electrochemical Properties of Electrode Materials

**Figure 6:**
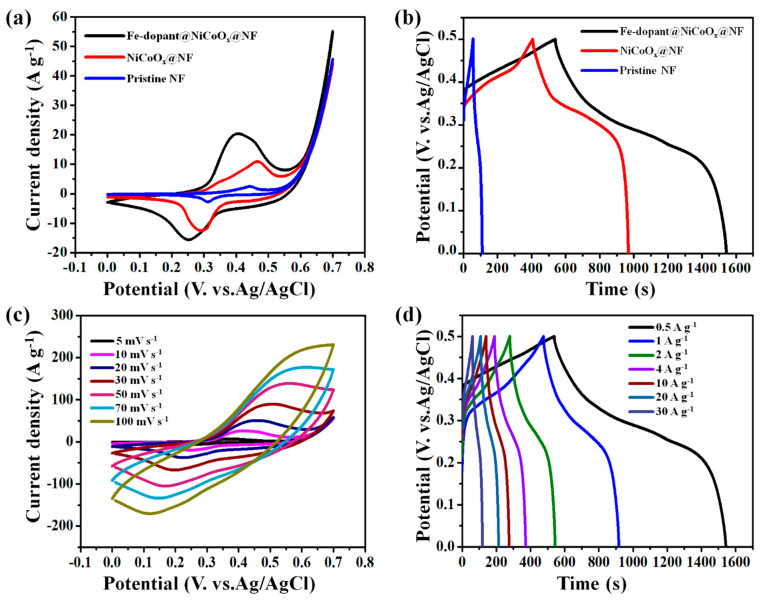
Electrochemical characteristic of the as-synthesized electrodes in a three-electrode system: (**a**) CV curves for NF, NiCoO_x_@NF nanowires, Fe-dopant@NiCoO_x_@NF nanoneedle composite electrodes at 10 mV·s^−1^; (**b**) GCD plots for NF, NiCoO_x_@NF nanowires, and Fe-dopant@NiCoO_x_@NF nanoneedles at 0.5 A g^−1^; (**c**) full CV tests for Fe-dopant@NiCoO_x_@NF nanoneedle composite electrodes at several applied scan rates; and (**d**) full GCD tests for Fe-dopant@NiCoO_x_@NF nanoneedle composite electrodes at several applied currents.

The CV and GCD tests of NF, NiCoO_x_@NF nanowires (Figure S1), and Fe-dopant@NiCoO_x_@NF nanoneedle composite electrodes were conducted using the setup of 3 electrodes in 3 M KOH aqueous solution. Figure 6a comprises the CV tests (plotted at 10 mV s^−1^) of NF, NiCoO_x_@NF nanowires, and Fe-dopant@NiCoO_x_@NF nanoneedle composite electrodes. A redox couples (oxidation–reduction peaks) can be clearly observed, indicating the Faradic type behavior of the supercapacitor electrode. It can be observed that the integral regions of CV plots for NF, NiCoO_x_@NF nanowires, and Fe-dopant@NiCoO_x_@NF nanoneedle composite electrodes successively increase, revealing that Fe-dopant@NiCoO_x_@NF nanoneedle composite electrodes have the greatest area specific discharges. To quantify the specific capacitance data, GCD analysis was successively performed from a 0 to 0.5 V potential window relative to Ag/AgCl. The GCD curves of NF, NiCoO_x_@NF nanowires, and Fe-dopant@NiCoO_x_@NF nanoneedle composite electrodes at 0.5 A g^−1^ are depicted in Figure 6b. Interstingly, the specific discharge capacitance of Fe-dopant@NiCoO_x_@NF nanoneedle composite electrodes (at 0.5 A g^−1^) is nearly twice that of the NiCoO_x_@NF nanowire electrodes and about triple that of the NF sample, indicating the superior storage charge ability and excellent energy storage performance of the as-grown composite. Thus, electrolyte ions would powerfully and suitably penetrate the inside surfaces of the active sample to prodcue the Faradic reaction.

The existence of an obvious pair of Faradic lines indicates the redox reactions and the lines are clearly shown in Figure 6c. As demonstrated in Figure 6c, the symmetric triangular formation of GCD curves exists at the greatest applicable current value of 30 A g^−1^, reflecting its notable Faradic reactions under the GCD charge mechanisms. As can be seen in Figure 6d, the storage mechanisms could be classified into two paths: the first one consists of the Faradic reaction contributions from the GCD effects, and the second path possesses the capacitance contribution reactions from the EDLC characteristic. The assistance of the Fe-dopant has been further studied by quantifying the GCD plots with numerous Fe amounts. The electrochemical storage mechanisms and the kinetic reactions were investigated by CV tests. Two pairs of redox species were obtained under the CV curves in the applied potential range of 0.0–0.5 V, which were mainly interconnected with the reversible Fe^3+^, Co^3+^/Co^2+^, and Ni^3+^/Ni^2+^ redox reactions [46,47,48]. Consequently, the electrochemical storage mechanisms of Fe-dopant@NiCoO_x_@NF nanoneedle composite samples can be explained via the equations [49,50,51]:

NiCo_2_O_4_ + H_2_O + OH− ↔ 2CoOOH + NiOOH + e− (2)

CoOOH + OH− ↔ CoO_2_ + H_2_O + e− (3)

Fe^3+^ + e− ↔ Fe^2+^ (4)

**Figure 7:**
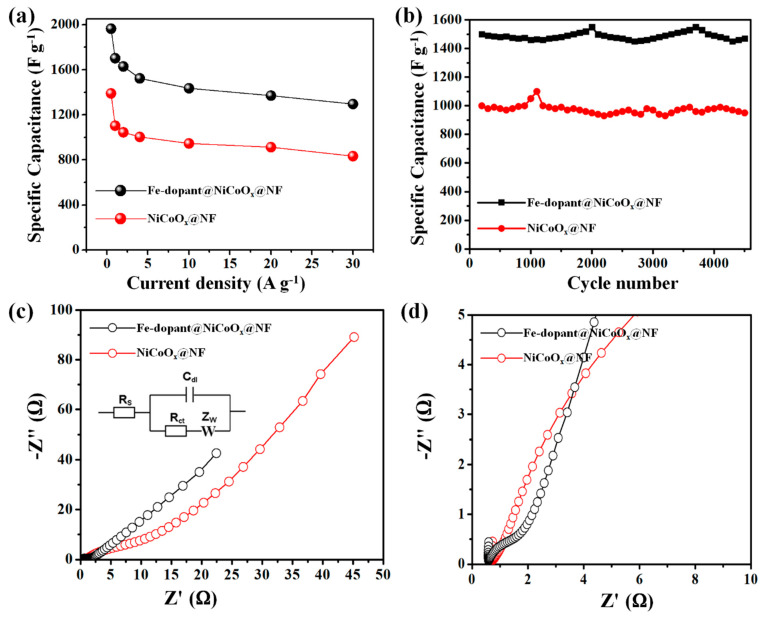
Electrochemical behavior of the as-developed electrodes in a three-electrode configuration: (**a**) Specific discharge capacity for as-fabricated NF, NiCoO_x_@NF nanowires, and Fe-dopant@NiCoO_x_@NF nanoneedle composite electrodes measured at diverse specific current, (**b**) cyclic stability properties, (**c**) EIS plots of the prepared electrodes (inset: fitted equivalent circuit), and (**d**) EIS plots of the enlarged high-frequency region.

The plots of GCD-specific discharge capacitances versus the specific current of the Fe-dopant@NiCoO_x_@NF and NiCoO_x_@NF samples are shown in Figure 7a. Apparently, the Fe-dopant@NiCoO_x_@NF nanoneedle composite electrodes achieve the largest discharge times at all current values compared with the binary NiCoO_x_@NF nanowire electrodes. The Fe-dopant@NiCoO_x_@NF nanoneedle composite exhibited a high specific capacitance of 1965 F g^−1^ at 0.5 A g^−1^, which is much larger than the values found in recent reports (shown in Table 1). When current values were increased from 0.5 to 30 A g^−1^, the capacitance of the Fe-dopant@NiCoO_x_@NF nanoneedle composite decreased from 1965 to 1289 F g^−1^, whereas the NiCoO_x_@NF nanowire electrodes achieved 1391 F g^−1^ at 0.5 A g^−1^. Particularly, the Fe-dopant@NiCoO_x_@NF nanoneedle composite achieved high rate capabilities (89.4%) when compared with pristine NiCoO_x_@NF nanowires (78.7%), indicating the effect of iron dopant on the electrical conductivity characteristics and the good construction of the Fe-dopant with NiCoO_x_ nanowires on NF.

Cycling stability is an additional crucial constituent for evaluating the electrochemical capabilities of our samples. Cycling stability was quantified under GCD tests at 4 A g^−1^ over 4500 long cycles (Figure 7b). Figure 7b depicts the cycling plots of NiCoO_x_@NF nanowires and Fe-dopant@NiCoO_x_@NF nanoneedle composite electrodes, respectively. It is detected that the Fe-dopant@NiCoO_x_@NF composite could be stable at 95.9% of the starting capacitance over 4500 cycles. On other hand, the capacitance of the NiCoO_x_@NF nanowire samples slightly diminishes to 82.6% of the prior data. Thus, hydrothermal preparations of Fe-dopant anchored on more stable NiCoO_x_ nanowires successively enhance the stability properties of composites, indicating excellent electrochemical stability behaviors.

EIS was further conducted to estimate the conductivity characteristics. All EIS plots displayed two principal categories. As seen in Figure 7c,d, the semi-circle diameters represent the charge transfer resistances (R_ct_), while the internal resistance, R_s_, is obtained from the intersection of Nyquist curves with the Z′-axis. Moreover, the angles in the lower frequency region determine the kinetic mass transfer procedures. The R_ct_ of NiCoO_x_@NF nanowires and Fe-dopant@NiCoO_x_@NF nanoneedle composite electrodes were approximated to be 0.57 and 0.36 Ω, respectively, revealing that Fe-dopant@NiCoO_x_@NF nanoneedle composites possess rapid kinetic charge transfers and low resistance internals. Such characteristics favor the paths for ions affected by the Fe-dopant particles to the internal zone of Fe-dopant@NiCoO_x_@NF nanoneedle composites [37,38]. Impressively, the Fe-dopant@NiCoO_x_@NF nanoneedle composites sample delivered a smaller circled line with possible vertical slope lines, indicating the ultra-fast rate of ion transport diffusion and adequate connections between electrolytes and electrode samples.

## 4. Conclusions

In summary, we have designed an earth-abundant, cost-effective transition metal pair composed of Fe-dopant and NiCoO_x_ nanowires for SC energy storage performance. The Fe-dopant with NiCo_2_O_4_ nanowires was effectively anchored and occupied on the NF skeleton, and the impact of synergistic reactions of greater redox activities was observed. Thus, the prepared Fe-dopant@NiCoO_x_@NF nanoneedle composite nanostructures resulted in the inclusion of energy storage active sites and enhanced charge transportation, and it lessened the transportation pathways for ion diffusion in the electrolyte. The outcomes revealed that Fe-dopant@NiCoO_x_@NF nanoneedle composites demonstrate notable electrochemical reactions as unique electrode materials for SCs. Consequently, the Fe-dopant@NiCoO_x_@NF nanoneedle composites exhibited a superior specific capacitance of 1965 F g^−1^ at 0.5 A g^−1^. In addition, the leading rate capability and the outstanding cycling stability were distinctly reflected under the capacitance retentions of 95.9% over 4500 cycles. Thus, preparing a unique nanostructure concept with transition metals will lead to progress in creating efficient SCs.

## Figures and Tables

**Table 1:** Specific capacitance comparisons between recent reports of transition metals and our highly efficient Fe-dopant@NiCoO_x_@NF nanoneedle composite electrodes in a 3-electrode configuration.

Electrode Materials	Synthesis Approach	Capacitance (F g^−1^)	Cycling Stability (No. of Cycles)	Ref.
NiCo_2_O_4_@PPy	Electrochemical deposition	1.44 F cm^−2^ (2 mA cm^−2^)	85% (5000)	[52]
NiCo_2_O_4_@Ni_0.85_Se	Hydrothermal	1454 (1 A g^−1^)	88.5% (10,000)	[53]
MWCNT/GO/NiCo_2_O_4_	Hydrothermal	707 (2.5 A g^−1^)	88% (5000)	[54]
NiCo_2_O_4_/graphene hydrogel/Ni foam	Electrochemical deposition	3.84 F cm^−2^ (2 mA cm^−2^)	92% (5000)	[55]
C/NiCo_2_O_4_	Hydrothermal	404 (1 A g^−1^)	87.1% (1000)	[56]
GE/NiCo_2_O_4_	Hydrothermal	591.5 (1 A g^−1^)	88.9% (2000)	[57]
NiCo_2_O_4_/GO	Hydrothermal	709.7 (1 A g^−1^)	84.7% (3000)	[58]
NiCo_2_O_4_ nanorods	Solvothermal	440 (5 mV s^−1^)	94% (2000)	[59]
NiCo_2_O_4_@MnO_2_	Hydrothermal	5.3 F cm^−2^ (1 mA cm^−2^)	90.1% (5000)	[60]
Fe-dopant@NiCoO_x_@NF	Hydrothermal	1965 F g^−1^ at (0.5 A g^−1^)	95.9% (4500)	This work

## Data Availability

No new data were created or analyzed in this study. Data sharing is not applicable to this article.

## Supplementary Materials

The following are available online at https://www.mdpi.com/article/10.3390/nano13020292/s1, Figure S1. Electrochemical characteristic of the as-synthesized electrodes at a three-electrode system: (a) Full CV tests for NiCoO_x_@NF nanowires electrode at everal applied scan rates, and (b) Full GCD tests for NiCoO_x_@NF nanowires electrode at several applied currents.
