# Supplementary material for: In Situ Grown Mesoporous Structure of Fe-Dopant@NiCoOX@NF Nanoneedles as an Efficient Supercapacitor Electrode Material
Source: Nanomaterials (Basel). 2023 Jan 10;13(2):292. doi: 10.3390/nano13020292 (PMC9866587; doi:10.3390/nano13020292)
Supplement: Supplementary file 1 [file nanomaterials-13-00292-s001.zip › nanomaterials-2113421-supplementary.pdf]

Supplementary Materials

In Situ Grown Mesoporous Structure of Fe-Dopant@NiCoOx@NF Nanoneedles as an Efficient Supercapacitor Electrode Material

Yedluri Anil Kumar [1,2,†], Ganesh Koyyada [3,†], Dasha Kumar Kulurumotlakatla [4], Jae Hong Kim [3], Md Moniruzzaman [5,*], Salem Alzahmi [1,2,*] and Ihab M. Obaidat [2,6,*]

[1] Department of Chemical & Petroleum Engineering, United Arab Emirates University, Al Ain-15551, United Arab Emirates; yedluri.anil@gmail.com

[2] National Water and Energy Center, United Arab Emirates University, Al Ain 15551, United Arab Emirates

[3] Department of Chemical Engineering, Yeungnam University, 214-1, Daehak-ro 280, Gyeongsan 712-749, Gyeongbuk-do, Republic of Korea; ganeshkoyyada@gmail.com (G.K.); jaehkim@ynu.ac.kr (J.H.K.)

[4] Graduate School of Convergence Science, Pusan National University, San 30 Jangjeon-dong, Geumjeong-gu, Busan 609-735, Republic of Korea; dashakumar1212@gmail.com

[5] Department of Chemical and Biological Engineering, Gachon University, 1342 Seongnam-daero, Seongnam-si, Gyeonggi-do 13120, Republic of Korea

[6] Department of Physics, United Arab Emirates University, Al Ain 15551, United Arab Emirates

* Correspondence: mani57chem@gachon.ac.kr (M.M.); s.alzahmi@uaeu.ac.ae (S.A.); iobaidat@uaeu.ac.ae (I.M.O.)

† These authors contributed equally to this work.

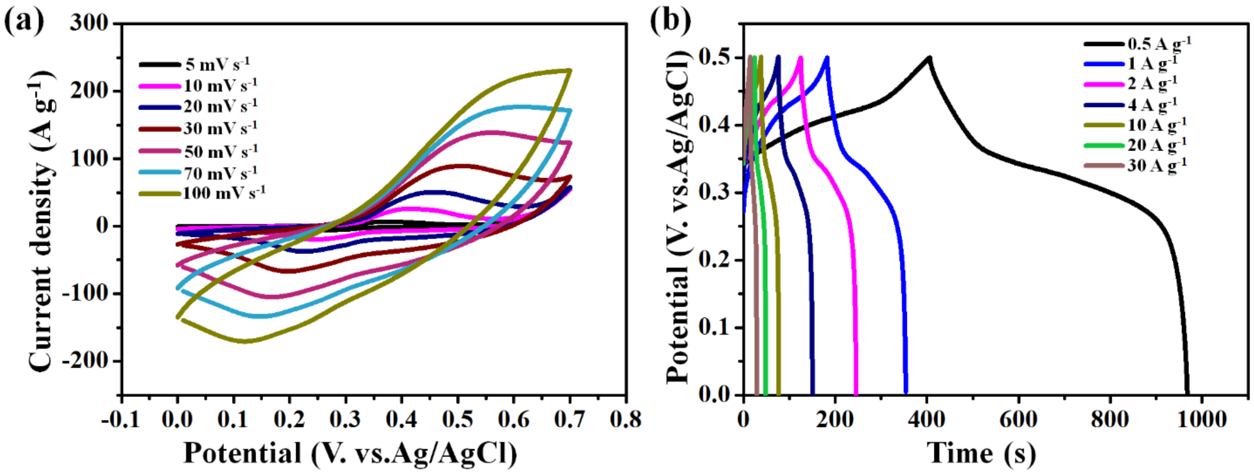

Figure S1. Electrochemical characteristic of the as-synthesized electrodes at a three-electrode system: (a) Full CV tests for NiCoOx@NF nanowires electrode at everal applied scan rates, and (b) Full GCD tests for NiCoOx@NF nanowires electrode at several applied currents.
